# Untargeted Metabolomics of Korean Fermented Brown Rice Using UHPLC Q-TOF MS/MS Reveal an Abundance of Potential Dietary Antioxidative and Stress-Reducing Compounds

**DOI:** 10.3390/antiox10040626

**Published:** 2021-04-19

**Authors:** Akanksha Tyagi, Su-Jung Yeon, Eric Banan-Mwine Daliri, Xiuqin Chen, Ramachandran Chelliah, Deog-Hwan Oh

**Affiliations:** Department of Food Science and Biotechnology, College of Agriculture and Life Sciences, Kangwon National University, Chuncheon 200-701, Korea; akanksha@kangwon.ac.kr (A.T.); sujung0811@gmail.com (S.-J.Y.); ericdaliri@kangwon.ac.kr (E.B.-M.D.); cxq20135331@gmail.com (X.C.); ramachandran865@gmail.com (R.C.)

**Keywords:** brown rice, fermentation, germination, antioxidants, stress, bioactive compounds, untargeted metabolomics, functional food, health benefits

## Abstract

Free radical-induced oxidative stress is the root cause of many diseases, such as diabetes, stress and cardiovascular diseases. The objective of this research was to screen GABA levels, antioxidant activities and bioactive compounds in brown rice. In this study, we first fermented brown rice with different lactic acid bacteria (LABs), and the best LAB was selected based on the levels of GABA in the fermentate. *Lactobacillus reuterii* generated the highest levels of GABA after fermentation. To ascertain whether germination can improve the GABA levels of brown rice, we compared the levels of GABA in raw brown rice (Raw), germinated brown rice (Germ), fermented brown rice (Ferm) and fermented-germinated brown rice (G+F) to identify the best approach. Then, antioxidant activities were investigated for Raw BR, Germ BR, Ferm BR and G+F BR. Antioxidant activity was calculated using a 2,2-diphenyl-1-picryl hydrazile radical assay, 2,2-azino-bis-(3-ethylene benzothiozoline-6-sulfonic acid) radical assay and ferric-reducing antioxidant power. In Ferm BR, DPPH (114.40 ± 0.66), ABTS (130.52 ± 0.97) and FRAP (111.16 ± 1.83) mg Trolox equivalent 100 g, dry weight (DW), were observed as the highest among all samples. Total phenolic content (97.13 ± 0.59) and total flavonoids contents (79.62 ± 1.33) mg GAE/100 g and catechin equivalent/100 g, DW, were also found to be highest in fermented BR. Furthermore, an untargeted metabolomics approach using ultra-high-performance liquid tandem chromatography quadrupole time of flight mass spectrometry revealed the abundance of bioactive compounds in fermented BR, such as GABA, tryptophan, coumaric acid, L-ascorbic acid, linoleic acid, β-carotenol, eugenol, 6-gingerol, etc., as well as bioactive peptides which could contribute to the health-promoting properties of *L. reuterii* fermented brown rice.

## 1. Introduction

Rice (*Oryza sativa*) is one of the most important and stable human food crops in the world. Rice grain is a major source of carbohydrate and protein, as well as other important nutrients, for billions of people worldwide, particularly in developing countries [1]. In recent years, brown rice (BR) has gained growing attention due to its obvious advantages over white rice for the content of bioactive compounds [2], as it is known that the wrong diet may lead to an imbalance between free radical formation and radical scavenging ability, causing oxidative stress [3].

A lower reactive oxygen species (ROS) concentration is important for normal cellular signaling, although higher concentrations and long-term exposure to ROS cause damage to cellular macromolecules such as DNA, lipids and proteins, eventually leading to necrosis and apoptotic cell death [4]. The normal and proper functioning of the central nervous system (CNS) depends entirely on the chemical integrity of the brain. It is well known that the brain absorbs a significant amount of oxygen and is very high in lipid content and is vulnerable to oxidative stress [5]. High oxygen consumption contributes to an unsustainable output of ROS. Besides, neuronal membranes are found to be rich in polyunsaturated fatty acids, which are particularly susceptible to ROS [5].

Nowadays, antioxidants from natural sources such as fruits, cereals and vegetables have become a profitable alternative to prevent oxidative stress. A variety of studies have identified various bioactive compounds in brown rice, such as phenolic acids, flavonoids, γ-oryzanol, aminobutyric acid (GABA), α-tocopherol and γ -tocotrienol, which contribute to the health-promoting properties of brown rice [6]. Additionally, gamma-aminobutyric acid (GABA) is a recognized non-protein amino acid. GABA is commonly recognized as one of the main brain neurotransmitters, and ingestion of GABA has been recognized as affecting many important physiological functions, including enhancing brain function, postponing intelligence loss [7] and relief of nervous stress [8].

Although polyphenols or bioactive compounds are not available only in free form, insoluble or bound polyphenols are part of the cell wall, while free polyphenols can be contained within the cell wall of the plant [9]. Bound polyphenols are connected by ester, ether or glycoside linkages to the cell wall components that increase the mechanical strength of the cell wall. The use of bioconversion processes like fermentation and germination has been established as an efficient method for releasing bound bioactive compounds (phenols, flavonoids, organic acids, etc.). Enzymes produced in these processes (hydrolyzing enzymes) or biological process enzymes can break down the bound bioactive compounds or the linkages between the cell wall components [10]. Lactic acid bacteria have been involved in fermenting food materials for a long time, to release polyphenols or bioactive compounds [11]. Moreover, germination also activates many dormant enzymes which induce the degradation of molecules by synthesis and respiration of new constituents of cells [11]. The potential antioxidant and beneficial properties of health-associated phenolic compounds found in brown rice varieties make them potential targets for functional food markets. Many fermented brown rice and germination brown rice products are also well recognized in the community because of their various beneficial effects on health.

Besides, metabolomics techniques like gas chromatography-mass spectrometry (GC-MS), ^1^H-nuclear magnetic resonance (^1^H-NMR) and liquid chromatography-mass spectrometry (LC-MS) have been used to classify food metabolites [12]. Among different commonly used techniques, LC-MS is the most widely used in metabolomics studies because of its sensitivity to detection, high resolution and non-derivatization of samples [13]. Based on the same LC-MS approach, ultra-high-performance liquid tandem chromatography quadrupole flight mass spectrometry (UHPLC-QTOF/MS) is a new approach in chromatography to evaluate and quantify further metabolites with more sensitivity [14].

Recently, there has been a significant rise in interest in the use of nutritious foods, rich in essential amino acids, polyphenols and bioactive compounds like GABA. Therefore, the present study sought to investigate antioxidant properties, including phenolic compounds, flavonoids and organic acids alongside the amino acid composition of brown rice using UHPLC-ESI-QTOF-MS/MS to explore the ability of bio convergence processes to be used as natural antioxidants and therapeutics for the development of functional foods.

## 2. Materials and Methods

### 2.1. Rice Samples

BR sample (*Oryza sativa* L. Variety Japonica) was purchased from the local market, Chuncheon, Gangwon-do, South Korea. Raw BR and after-processing (germination) BR was ground into a fine powder using an electric mill and sifted through mesh 40. Samples were stored at −20 °C before further extraction.

### 2.2. Chemicals and Cultures

All chemical reagents were of analytical grade. Acetonitrile, ethanol, acetone, methanol, sodium carbonate, sodium hydroxide, anhydrous sodium acetate, hydrochloric acid, potassium persulfate, acetic acid and sulfuric acid were purchased from Daejung Chemicals and Metals Co., Ltd., South Korea. The phenolic standards and other chemicals like Folin–Ciocalteu reagent, Trolox (6-hydroxy-2,5,7,8-tetramethylchroman-2-carboxylic acid), DPPH (2,2-diphenyl-1-picrylhydrazyl); ABTS (2,2′-Azino-bis (3-ethylbenzothiazoline-6-sulfonic acid), TPTZ (2,4,6-Tris(2-pyridyl)-s-triazine) and gallic, ferulic, caffeic, o-Coumaric and p-Coumaric acids were obtained from Sigma, South Korea.

*Lactobacillus reuterii* AKT1 and all other strains used in our study were obtained from the Department of Food Science and Biotechnology, Kangwon National University, Korea, to be used for fermentation. The bacteria stock culture was stored at −80 °C, in MRS broth (Difco), containing 20% glycerol (*v*/*v*).

### 2.3. Sample Preparation

#### 2.3.1. Brown Rice Germination

Brown rice (BR) germination was carried out by the method of [15] Cáceres et al. (2017), with some modification. Briefly, BR seeds (50 g) were washed 3–4 times with distilled water, 0.2% of sodium hypochlorite solution (1:5 *w*/*v*) as a disinfectant for 20 min at room temperature, and then rinsed thoroughly with distilled water. Drained BR was then soaked in distilled water for 12 h at 28 ± 1 °C. The soaking solution was changed every 6 h. After soaking, germination was carried out at a constant temperature (DAIHAN LABTECH. Co., Ltd., Incubator, Gyeonggi-do, Korea) at 30 ± 1 °C and 85% relative humidity in the dark for 48 h. Following germination, the germinated brown rice (GBR) was dried in a blast drying oven (OF-22GW, JEIO Tech Instrument Co., Ltd., Seoul, Korea) at 55 °C for 6 h (Figure S1). After drying, the GBR was ground into a fine powder using an electric mill and sifted through mesh 40.

#### 2.3.2. Brown Rice Fermentation

Ground brown rice powder (10 gm/10 mL) was mixed with distilled water, sterilized in an autoclave at 121 °C for 15 min and then left until cool. Then, approximately 5 mL of 2 × 10^7^ cfu/mL spores suspension^−1^ of 10 different lactic acid bacteria strains (*P. pentosaceus* (FMC1), *L. fermentum* (FMF2), *L. fermentum* (AKT2), *L. rhamnosus* (FMR1), *L. rhamnosus* (FMR2), *L. brevis* (FMB1), *L. brevis* ATCC (STANDARD), *L. plantarum* (FMP1), *L. plantarum* (FMP2) and *L. reuterii* (AKT1)) was obtained from actively growing slants in sterile water, which was then inoculated into sterilized (autoclaved) BR samples and incubated at 37 °C with 150 rpm agitation for 48 h. After 48 h of fermentation, the media were then centrifuged at 10,000× *g* for 10 min and the supernatant was freeze-dried and stored at −20 °C until further studies.

#### 2.3.3. Preparation of Ethanolic Extracts

Extraction was carried out using a procedure of Shao-Hua et al. (2012) [16], with some modification. Brown rice powder (1 gm) was mixed with 20 mL of 50% ethanol (1:20 *w*/*v*) for 4 h in an electric shaker (RK-2D, DAIHAN scientific, Korea) at 50 °C. After that, extracts were centrifuged (Union 32R plus, Hanil Science Industrial, Korea) and the supernatant was collected at 4000× *g* for 10 min and the residue was re-extracted twice under the same conditions. The supernatants were filtered (0.20 μm Whatman™ syringe filter, Lk Lab Korea), evaporated at 50 °C and stored at −20 °C for further use. A stock solution of the sample was prepared with a concentration of 1 mg/mL. This was the stock that was used for the entire analysis.

### 2.4. Detection of Gamma-Aminobutyric Acid (GABA)

GABA content of the 50% ethanolic extract of raw rice, fermented rice, germinated brown rice (GBR) and germination combined with fermentation was determined as reported by Tiebing Liu et al. (2015) [17], with some modification. A Poroshell HPH-C18 column (4.6 × 100 mm, 2.7 μm) using pre-column derivatization with dansyl chloride by HPLC was used for GABA detection. The derivatization method was as follows: 0.2 mL of GABA standard solution, as well as rice samples (1 mg/mL), were mixed with the same volume, 0.2 mL, of dansyl chloride solution in a 1 mL brown volumetric flask and shaken. Then, samples were placed in a 50 °C water bath (VS-1205W, Vision Scientific Co. Ltd., Korea) for 50 min away from light, cooled to room temperature and diluted to a volume of 1.0 mL with methanol. Then, the solution was filtered through filters of 0.45 μm. The pH of the samples was adjusted to 8–8.5 before derivatization by adding 0.1 mol/L sodium bicarbonate.

The HPLC conditions were as follows: HPLC (Agilent Technologies, Baudrats, Germany) with an Infinity Lab Poroshell HPH-C18 column (4.6 × 100 mm, 2.7 μm) fitted with a guard column (Agilent Technologies, CA, USA); a flow rate of 1 mL/min; a column temperature of 30 °C; phase A, methanol; mobile phase B, water; and an amount of injection of approximately 2 μL.

### 2.5. Total Phenolic Content (TPC)

TPC was measured using the Folin–Ciocalteu colorimetric method mentioned formerly by Chang et al., (2018) [18] with some modification. Briefly, for a short duration of 6 min, 100 μL of sample extract or standard solution of gallic acid was treated with 200 μL of Folin–Ciocalteu reagent. The mixture was then alkalized with 1 mL of 700 Mm Na_2_CO_3_. After being kept dark for 90 min, the absorbance was measured at 760 nm using the SpectraMax i3 plate reader (Molecular Devices Korea, LLC). The total phenolic content was determined from the standard gallic acid curve and expressed as milligrams of gallic acid equivalent per 100 g of sample (mg GAE/100 g).

### 2.6. Total Flavonoid Content (TFC)

The total flavonoid content (TFC) of ethanol extracts was calculated using the 24-well microplate method as defined by Bah et al. (2016) [19], with some modifications. Briefly, 200 μL of our sample extracts were added to 1 mL of distilled water and 75 μL of NaNO_2_ (50 g L^−1^). After 5 min of incubation, 75 μL of AlCl_3_ (100 g L^−1^) was applied to the reaction mixture. Later, after 6 min, 600 μL of distilled water and 500 μL of 1 M NaOH were added. The absorbance was read at 510 nm by using the SpectraMax i3 plate reader (Molecular Devices Korea, LLC). Catechin was used as a baseline and the results were expressed as milligram catechin equivalents per 100 g of sample (mg CE/100 g).

### 2.7. Antioxidant Assays

#### 2.7.1. DPPH Radical Scavenging Activity

DPPH radical scavenging activity assay was determined following the method of Chen et al. (2016) [20], with some modification. Briefly, a 24-well microplate was used with 200 μL sample extracts, which were mixed with 2 mL of freshly formulated 100 μM DPPH radical solution (4 mg DPPH in 100 mL 95 % *v*/*v* methanol). After 60 min of incubation at room temperature in the dark, the absorbance was measured at 515 nm using a spectrophotometer. The Trolox concentration plot with DPPH radical scavenging activity was used as a standard curve. DPPH values were expressed as mg Trolox equivalent (TE) per 100 g of sample DW.

#### 2.7.2. ABTS Radical Scavenging Activity

ABTS activity was measured as described by Ofosu et al. (2020) [21] with some modification. ABTS radical cation reagent was formed by the reaction of 7 mmol/L ABTS stock solution with 2.45 mmol/L potassium persulfate stock solution (1:1, *v*/*v*). The mixture was kept in the dark for 12–16 h at room temperature before use. The ABTS^•+^ reagent was then mixed with methanol and the absorbance was modified to 0.700 ± 0.020 at 734 nm. In 1 mL of ABTS radical solution, 100 μL of properly diluted crude extracts or standards were mixed. After 30 min of incubation at room temperature in the dark, the spectrophotometer (SpectraMax i3 plate reader (Molecular Devices Korea, LLC) was used to measure the absorbance at 734 nm. Trolox concentration with ABTS radical scavenging rate was used as a reference curve. ABTS values were expressed as mg Trolox equivalent (TE) per 100 g of sample DW.

#### 2.7.3. Ferric-Reducing Antioxidant Power (FRAP)

Ferric-reducing antioxidant strength was analyzed using the method as documented by Zeng et al. (2019) [22], with some improvements. Briefly, 0.1 mL of extract was combined with a FRAP reagent of 3.9 mL (FRAP reagent was prepared by using 50 mL acetate buffer (0.3 M, pH 3.6), 5 mL Tripyridyl triazine (TPTZ) solution (10 mM TPTZ in 40 mM HCl) and 5 mL FeCl_3_·6H_2_O (20 mM)), and set at 37 °C for 10 min, then the absorbance was performed at 593 nm. The findings were expressed as mg TE/100 g, DW.

### 2.8. UHPLC Q-TOF-MS/MS Identification

Ultra-high-performance liquid tandem chromatography quadrupole flight mass spectrometry (UHPLC Q-TOF-MS/MS) was used for the identification of bioactive compounds in rice. A rice sample (1 mg/mL) prepared after ethanol extraction was used for the analysis. The samples were centrifuged at 12,000× *g* for 15 min at 4 °C. Aliquots (1 mL) of the supernatants were filtered through 0.25 m pore size Millex syringe filters (Merck KGaA, Darmstadt, Germany) and transferred to LC-MS vials. The mass spectrometric analysis was conducted in both positive (ESI+) and negative (ESI-) ion modes. UHPLC (SCIEX ExionLC AD machine, MA, USA) fitted with a controller, AD pump, degasser, AD autosampler, AD column oven and photodiode array (PDA) detector (ExionLC), coupled with a quadrupole time-of-flight mass spectrometer (Q-TOF-MS) (X500R QTOF), was used for UHPLC and mass spectrometric analyses (LC-MS^2^). The protocol of Daliri et al. (2020) [23] was used in the detection method with some modifications. Briefly, the analytical column used was a 100 × 3 mm Accucore column C-18 (Thermo Fisher Scientific, Waltham, MA, USA). The sample (10 μL) was injected by an autosampler and eluted through a binary mobile phase column consisting of solution A, which was water containing 0.1% formic acid, and solution B, which was methanol. A flow rate of 0.4 mL/min was used with a linear gradient which was programmed for 25 min as follows: 0–3.81 min, 9 to 14% B; 3.81–4.85 min, 14 to 15% B; 4.85–5.89 min, 15% B; 5.89–8.32 min, 15 to 17% B; 8.32–9.71 min, 17 to 19% B; 9.71–10.40 min, 19% B; 10.40–12.48 min, 19 to 26% B; 12.48–13.17 min, 26 to 28% B; 13.17–14.21 min, 28 to 35% B; 14.21–15.95 min, 35 to 40% B; 15.95–16.64 min, 40 to 48% B; 16.64–18.37 min, 48 to 53% B; 18.37–22.53 min, 53 to 70% B; 22.53–22.88 min, 70 to 9% B; and 22.88–25.00 min, 9% B. For positive ion mode, the capillary temperature and voltage were set at 320 °C and 40 V, respectively, the sheath gas flow was set at 40 arb. units and an aux gas flow of 8 arb. units, spray voltage at 3.6 kV and tube lens voltage at 120 V. In negative ion mode, capillary temperature and voltage were set at 320 °C and −40 V, respectively, sheath gas flow at 40 arb. units and gas flow to 8 arb. units, spray voltage up to 2.7 kV and tube lens voltage up to −120 V. In both positive and negative ion modes, the study was carried out using the Fourier transform mass spectrometry (FTMS) full scan ion mode, applying a mass scan range of 115–1000 *m*/*z* and a resolution of 30,000 full width at half maximum (FWHM) while the spectra were acquired in centroid mode. The scanning time under these conditions was approximately 1 s.

### 2.9. Statistical Analysis

The data was analyzed using Graphpad Prism 8.0. Differences in mean values between differently treated brown rice samples phenolic extracts were calculated using one-way variance analysis (ANOVA) followed by a Tukey and Duncan test at *p* < 0.05 significance stage using the SPSS program and Graphpad Prism 8.0. The findings were described as average ± standard deviation (SD).

Compound identification was achieved by using empirical formula finders like Pubchem (https://pubchem.ncbi.nlm.nih.gov/) (access on 9 April 2021) or ChemSpider (http://www.chemspider.com/) (access on 9 April 2021). ClustVis program (http://biit.cs.ut.ee/clustvis/) (access on 17 April 2021) and Paleontological Statistics (PAST) was used in multivariate statistical analyses, including main component analysis (PCA) and heat maps [24]. PCA was used to compare the changes among brown rice samples. Heat maps were drawn using peak areas of samples by clustVis [25].

## 3. Results and Discussion

### 3.1. Detection of Gamma-Aminobutyric Acid (GABA)

**Figure 1:**
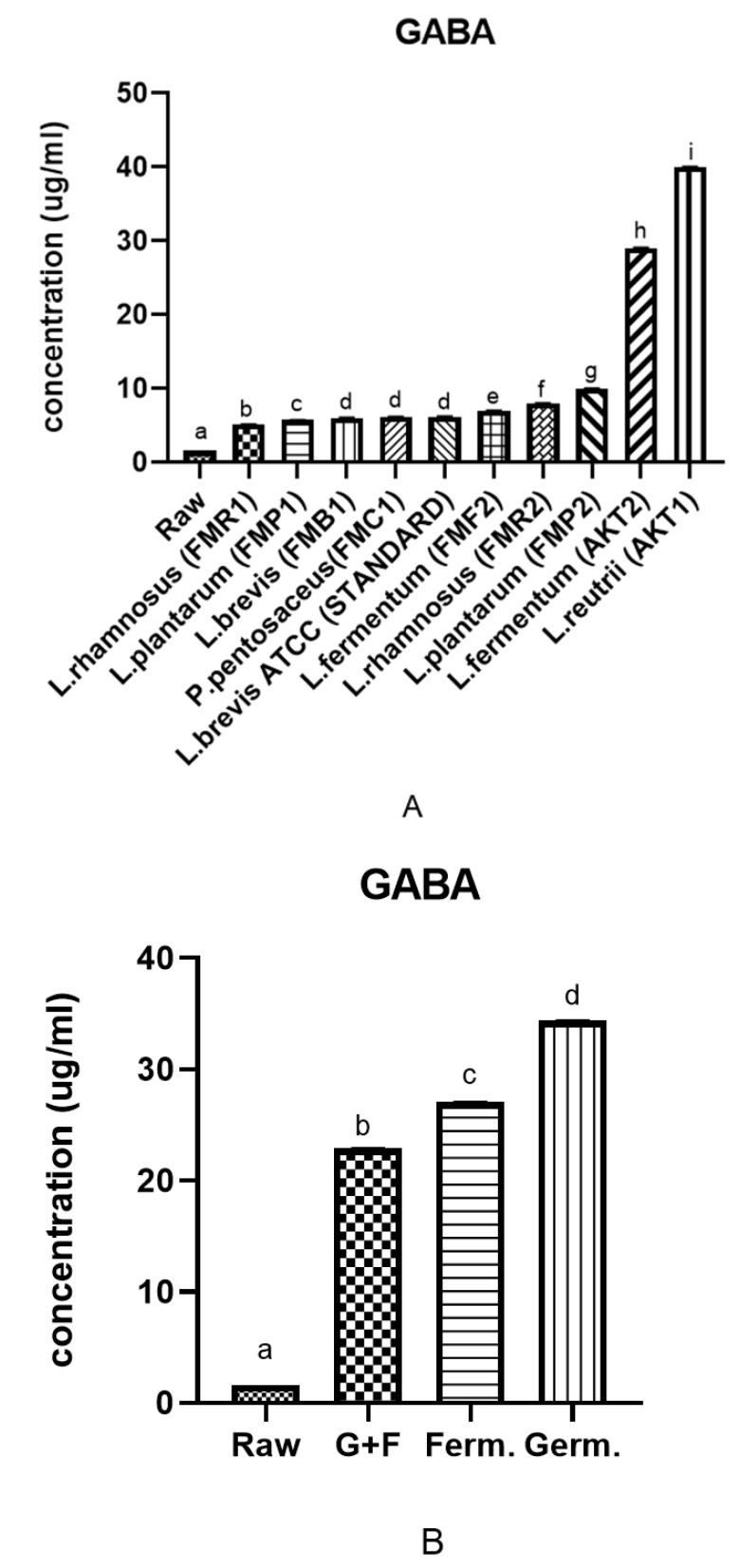
(**A**) Screening of different *Lactobacillus* strains (48 h fermented). (**B**) Comparison of GABA content among raw, germination (Germ), fermentation (Ferm) and germination combined with fermentation (G+F) treatments. All values are expressed as the mean ± SD of triplicate experiments. The sample concentration used was 1 mg/mL, and a–i superscripts with different letters indicate a significant difference, while sample superscript letters indicate no significant difference (*p* < 0.05) found by using SPSS and Graphpad Prism 8.0 programs.

GABA, the main inhibitory neurotransmitter, has multiple physiological functions such as being a blood pressure regulator, cardiovascular disease defender and hormonal and cell regulator. It is also associated with brain and mental disorders [26]. Therefore, we first screened different fermentation bacteria (LABs) for GABA concentration (Figure 1A), as it is linked to stress-related disorders. Among 10 lactobacillus strains, *L. reuterii* (AKT1) showed the highest GABA concentration in HPLC detection (Table S1). All the results are expressed as the mean ± SD of triplicate analyses, and additional statistical analyses were carried out using they Tukey and Duncan tests, *p* ≤ 0.05. These results show *L. reuterii* as the most efficient bacteria in GABA production during fermentation among all other LABs used for screening. In raw rice, GABA content was found to be 1.61 ± 0.001 μg/mL, whereas in *L. reuterii* fermented brown rice it was 27.03 ± 0.055 μg/mL. In this research, we found a higher GABA concentration than reported by Cáceres et al. and Le et al. [27,28].

After screening fermentation bacteria, we compared fermentation (Ferm) with germinated brown rice (Germ) and the germination combined with fermentation approach (G+F). In germinated samples, the GABA content was 34.43 ± 0.026, and in combination (G+F) samples it was found to be 22.91 ± 0.028 (Figure 1B). Germination was found to be higher than fermentation, so further analysis was carried out to find out more about the bioactive compounds produced during different bio convergence approaches in brown rice. We performed germination for 24, 48 and 72 h, and 48 h germination was found to be the best among GABA screenings. We found no difference in GABA concentration after 48 h and an increase in GABA content after 24 h, so 48 h was selected for further analysis (24 and 72 h data not shown). Fermentation was also carried out for 48 h to compare the same duration of fermentation and germination.

### 3.2. Total Phenolic Content and Total Flavonoid Content of Ethanol Extracts

Phenolics are a class of phytochemicals that have one or more hydroxyl groups of aromatic rings, and phenolic content has been linked to the antioxidant effects of grains [29]. Phenolic-rich foods have high antioxidant potential and may counteract the negative effects of pro-inflammatory and pro-oxidative foods. The TPC and TFC of all four samples are shown in Table 1 as the mean ± SD of triplicate analyses with statistically significant differences (*p* ≤ 0.05). TPC was found to be lowest in raw brown rice samples, 16.75 ± 0.75 mg GAE/100 g, DW, and highest in *L. reuterii* AKT1 fermented brown rice samples, 97.13 ± 0.59 mg GAE/100 g, DW. Hydrolysis by enzymes in germination and fermentation usually enhances the total phenolic content and this was also observed in this study. TPC increased with germination (74.70 ± 0.72 mg GAE/100 g, DW) and fermentation (97.13 ± 0.59 mg GAE/100 g, DW) as compared with the raw BR sample. This study shows a higher TPC content of brown rice than reported by [30,31].

Flavonoids are a group of phenolic compounds consisting of two aromatic rings (A and B rings) bound together by a three-carbon structure generally found in an oxygenated heterocyclic ring (C ring). They have potent antioxidant activity and are associated with a reduced risk of chronic diseases. Total flavonoid content was found to be higher in fermented brown rice, 79.62 ± 1.33 mg catechin equivalent 100 g, DW, followed by the combined approach (G+F BR), germination and raw BR (Table 1). TFC content was lower than TPC in brown rice samples. The total TFC in this study was found to be higher than reported by Huang et al. [16], similarly to TPC, but both TPC and TFC were found to be lower than reported by Gong et al. [32]. These differences in brown rice values from different researchers may be due to genotype, cultivation landscape and climate conditions [33]. Besides, it is worth noting that the phenolic content can be significantly influenced by different extraction solvents and procedures.

### 3.3. Antioxidant Assay (DPPH, ABTS, FRAP)

Antioxidants are exogenous or endogenous molecules that reduce any form of oxidative/nitrosative stress or its consequences. Phenolic compounds are generally considered to be desired components for human health, due to their antioxidant activity. Various mechanisms, such as free radical scavenging, reducing capacity, metal ion chelation and lipid peroxidation inhibition, have been studied to explain how rice extracts could be used as effective antioxidants [34]. In recent years, germination and fermentation processes have also been considered to be an effective approach for enhancing antioxidant activities in cereals. In the present study, DPPH, ABTS and FRAP assays were used to evaluate the antioxidant activity of different treated brown rice samples. The antioxidant values of DPPH, ABTS and FRAP of phenolic extracts of BR are presented in Table 1.

One of the most frequently used assays in the determination of antioxidant activity in natural products is the measurement of the scavenging activity of the 2,2′-diphenyl-1-picrylhydrazyl (DPPH) radical by spectrophotometry. The DPPH radical scavenging activity was highest in the fermented brown rice sample (*L. reuterii* AKT1) (114.40 ± 0.66 mg Trolox equivalent 100 g, DW), followed by G+F BR and germinated brown rice samples (105.99 ± 0.59 and 89.60 ± 1 mg Trolox equivalent 100 g, DW). The lowest values were found in raw BR (25.653 ± 0.98 mg Trolox equivalent 100 g, DW).

Similarly, in in-plant materials and grains, ABTS is an important method for the determination of radical scavenging activity. In addition, the FRAP assay was originally designed to assess plasma antioxidant ability, but it has also been commonly used in a wide variety of pure compounds and biological samples to determine antioxidant capacity. The test operates on the principles of antioxidant donation of electrons. It measures changes in absorption as a result of the formation of blue iron (II) from colorless iron oxide (III). In our research, the same trend with DPPH was observed in ABTS and FRAP assays. ABTS activity was measured to be highest in fermented BR (130.52 ± 0.97 mg Trolox equivalent 100 g, DW), followed by G+F and germination (Table 1). In ABTS the lowest activity was from raw BR. Similarly, ferric-reducing antioxidant power (FRAP) was the highest in fermented BR (111.16 ± 1.83 mg Trolox equivalent 100 g, DW) followed by G+F and germination. A correlation was observed among the trends in all antioxidant assays. These findings were found to be higher than in earlier reports by Lin et al. (2019) [35] and IIowefah et al. (2017) [30] in fermented BR and Cho et al. (2018) [36] for germinated BR. Besides, other studies have reported the high antioxidant activity of brown rice, such as Rao et al. [37].

### 3.4. UHPLC Q-TOF-MS/MS Detection

Ultra-high-performance liquid tandem chromatography quadrupole flight mass spectrometry (UHPLC-Q-TOF-MS/MS) detection is considered the gold standard technique for the precise detection and quantification of a wide variety of components. Therefore, in this study, we used this detection technique for the identification of bioactive compounds in brown rice.

#### 3.4.1. Amino Acid Level in Brown Rice

**Figure 2:**
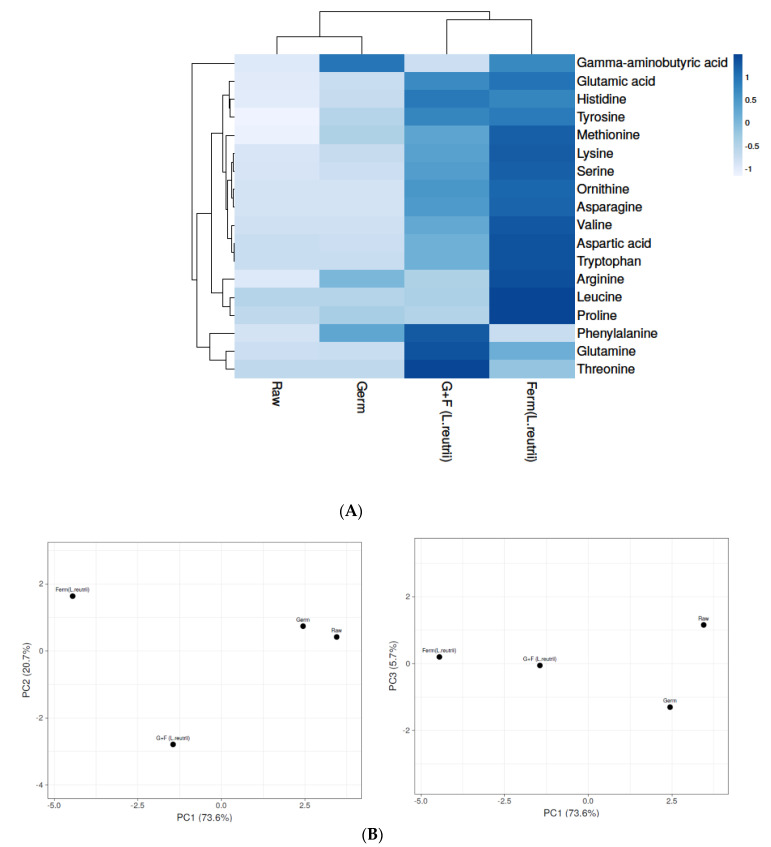
Levels of amino acids in raw, germinated, fermented (*L. reuterii* AKT1) and G+F (*L. reuterii* AKT1) samples. (**A**) The heat map shows different levels of amino acids in samples based on shades of blue. (**B**) Identification of principal component analysis (PCA) of Raw, Germ, Ferm (*L. reuterii*) and G+F samples was shown by comparing PC1 with PC2 and PC 1 with PC3. Germ-germinated brown rice, Ferm- fermented brown rice, G+F-germinated + fermented brown rice.

In the growth and development of organisms, amino acids play an important role and can also improve the taste of food. In this study, a total of eighteen amino acids were detected in brown rice (Figure 2A, Table S2) and differently treated samples showed statistically significant differences from each other after comparing levels of amino acids (ANOVA Table S2 *p* ≤ 0.05). Raw brown rice contained the least number of amino acids, whereas germination and fermentation lead to an increase in amino acid content. The levels of amino acids were detected to be highest in the *L. reuterii* AKT1 fermented BR sample, which is potentially because during fermentation microorganisms produce enzymes that lead to the formation of several metabolites and bioactive compounds from the food matrix [38]. Additionally, an increase was also observed in the germination process due to the activation of many dormant enzymes which enhance bioactive compounds in rice, whereas in the raw sample, a low level might be the reason for more bound amino acids with parent molecules. In the fermented sample, levels of some essential amino acids like methionine, histidine, valine, tryptophan, leucine and lysine increased drastically after fermentation, but not phenylalanine and threonine. Additionally, certain conditionally essential amino acids were also found to be higher in fermented brown rice samples (ornithine, serine, arginine, proline, tyrosine and glutamine) (Figure 2A). Moreover, the G+F approach also enhanced the level of amino acids as compared with the germination-only approach. Principal component analysis (PCA) was applied on amino acids to see the diversity and correlation among samples, and the raw BR profile was found to be correlated with the germinated sample but divergent from the fermented BR and G+F BR samples, whereas after comparing PC1 with PC3, the G+F BR sample showed some correlation with the fermented (*L. reuterii*) BR sample, as shown in the PCA graph (Figure 2B) (Table S2). PCA analysis showed the same trend as was observed in the heat map analysis.

#### 3.4.2. Phenolic Compounds in Brown Rice Samples

**Figure 3:**
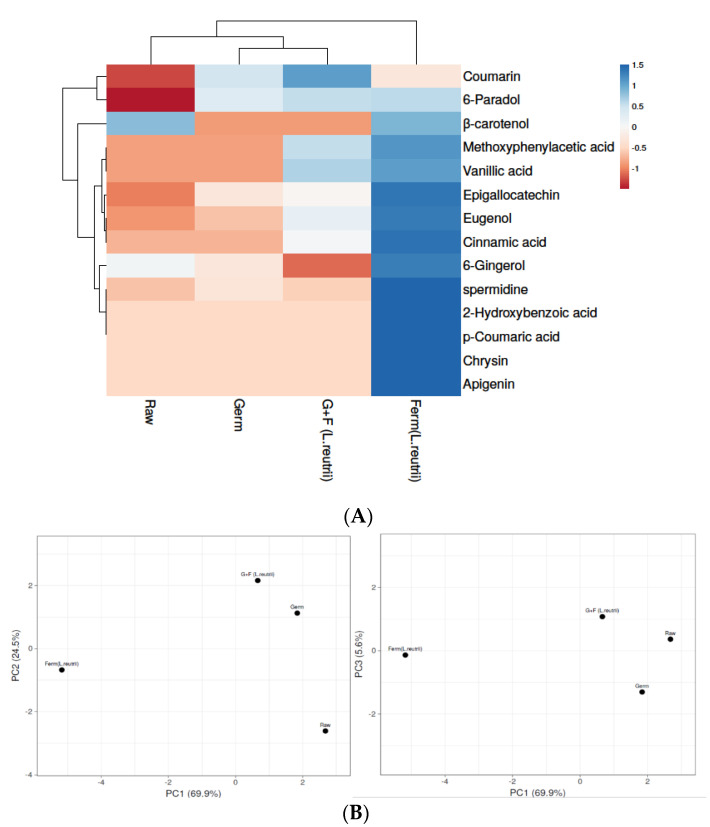
Levels of phenolic compounds in raw, germinated, fermented (*L. reuterii* AKT1) and G+F (*L. reuterii* AKT1) samples. (**A**) The heat map shows different levels of phenolic compounds in samples based on colors from blue to red, representing the level of phenolic compounds in decreasing order. (**B**) Identification of principal component analysis (PCA) of Raw, Germ, Ferm (*L. reuterii*) and G+F samples was shown by comparing PC1 with PC2 and PC1 with PC3. Germ- germinated brown rice, Ferm- fermented brown rice, G+F-germinated + fermented brown rice.

Fourteen phenolic compounds were identified in brown rice samples by UPLC-ESI-Q-TOF-MS/MS. Phenolic compounds have been suggested to account for the health benefits of rice intake in the prevention of chronic diseases, including cardiovascular disease, type II diabetes, obesity, oxidative stress, stress-related disorders and cancer [39]. The contents of phenolic compounds in different processed brown rice samples are presented in Table S3 and significant differences were observed after comparing the levels of each sample (*p* ≤ 0.05). Heat map analysis was used for clustering phenolic compounds based on their concentrations (Figure 3A) where the color scheme from blue to red shows concentration in decreasing order. The results show that the highest phenolic compounds were detected in the fermented (*L. reuterii* AKT1) brown rice sample, followed by G+F (*L. reuterii* AKT1), germinated and raw. Phenolic compounds usually occur in an esterified form linked to the cereal wall matrix in the cereals, as they are not readily available [40]. Fermentation is considered to be a possible strategy not only to increase AAs but also to release insoluble bound phenolic compounds and thus to improve the poor bioavailability of grain phenolics [41]. In the present study, many phenolic compounds such as β-carotenol [42], eugenol [43], apigenin [44], 6-gingerol [45], cinnamic acid [46] and vanillic acid [47] were detected in high concentrations in fermented brown rice, and all of these have been reported as strong antioxidants, as well as able to reduce stress-related behaviors. Principal component analysis (PCA) was applied for better interpretations, and to avoid multicollinearity (Figure 3B) (Table S3). In PCA, fermented (*L. reuterii* AKT1) brown rice was found to be divergent from all other samples, as it contained the highest concentration of phenolic compounds.

Comparing different approaches, we agree with the present reported studies that fermentation increases phenolic compounds as well as the bioavailability and bio-accessibility of cereal grains like brown rice [48].

#### 3.4.3. Organic Acid Level in Brown Rice Samples

**Figure 4:**
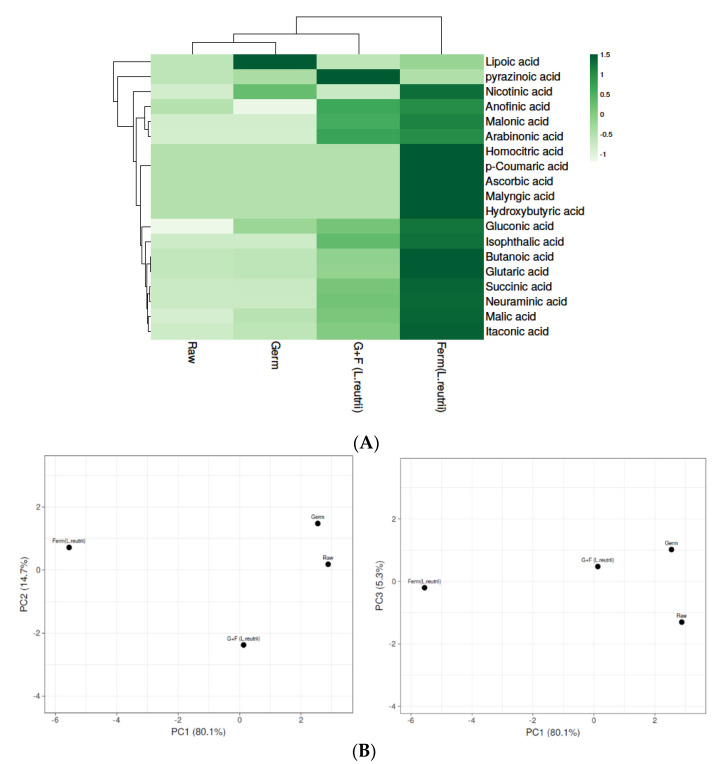
Levels of organic acids in Raw, Germinated, Fermented (*L. reuterii* AKT1) and G+F (*L. reuterii* AKT1) samples. (**A**) The heat map shows different levels of organic acids in samples based on colours as green to white represents the level of organic acids in decreasing order. (**B**) Identification of principal component analysis (PCA) of Raw, Germ, Ferm (*L. reuterii* AKT1) and G+F samples was shown by comparing PC1 with PC2 and PC1 with PC3. Germ- germinated brown rice, Ferm- fermented brown rice, G+F- germinated + fermented brown rice.

In the present study, nineteen organic acids, including lipoic acid, pyrazinoic acid, anofinic acid, malonic acid, arabinonic acid, nicotinic acid, homocitric acid, p-coumaric acid, ascorbic acid, malyngic acid, hydroxybutyric acid, gluconic acid, isophthalic acid, succinic acid, neuraminic acid, malic acid, itaconic acid, butanoic acid and glutaric acid, were detected in the tested brown rice samples (Table S4). Additionally, as shown in Table S4, organic acid levels were found to be significantly different in differently treated samples (*p* ≤ 0.05). Heat map analysis was used for clustering brown rice samples based on their organic acid concentration in differently treated samples (Figure 4A). The levels of organic acids were enhanced after germination and fermentation treatments, leading to the highest organic acids content in the *L. reuterii* AKT1 fermented brown rice sample (Table S4). Organic acids like ascorbic acid [49], p-coumaric acid [50], hydroxybutyric acid [51] and lipoic acid [52] were widely reported as strong antioxidants, as well as in the improvement of stress-related disorders in various studies. Fermentation triggers the structural degradation of the cell wall via the metabolic activities of microbes, leading to the synthesis of different bioactive compounds [53]. The functions of proteases, amylases and xylanases derived from fermenting microorganisms and cereal grains also contribute to grain modification and to the distortion of chemical bonds that, subsequently, release bound bioactive compounds. Additionally, during germination, due to the respiration and synthesis of new cell constituents, the degradation of certain molecules leads to bioactive components that enhance the functional and nutritional properties of germinated rice [54]. Principal component analysis (PCA) was applied to group organic acids that were more strongly correlated to avoid multicollinearity for the better elucidation of results (Figure 4B) (Table S4). The organic acid profile of fermented brown rice was found to be divergent from the other three samples, similar to the heat map analysis. Whereas raw BR and germinated BR samples were found to be more strongly correlated with each other in PC1 with PC2 analysis, G+F samples were found to be somehow correlated with Germ BR in PC1 with PC3 analysis.

#### 3.4.4. Fatty Acid Composition in Brown Rice Samples

**Figure 5:**
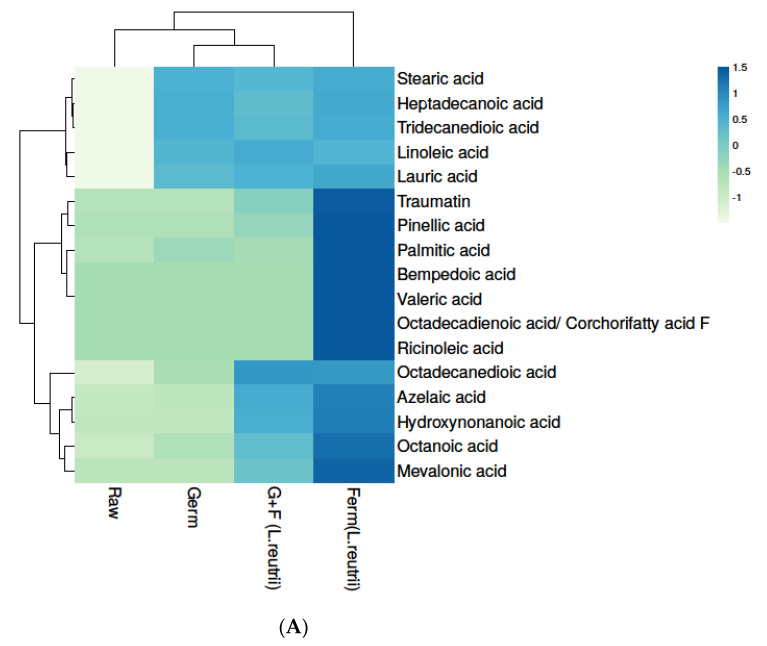

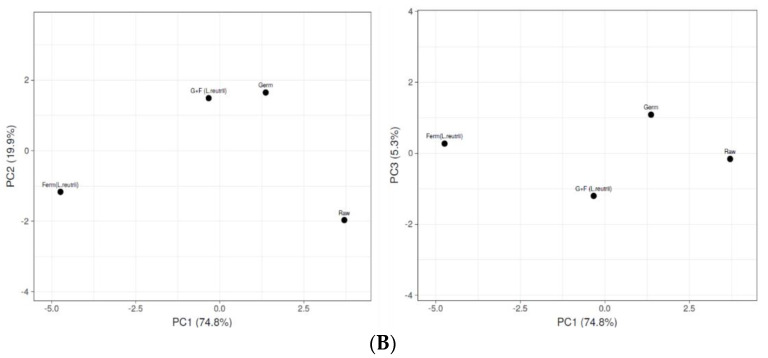
Levels of fatty acid in raw, germinated, fermented (*L. reuterii* AKT1) and G+F (*L. reuterii* AKT1) samples. (**A**) The heat map shows different levels of fatty acid in samples based on colors, as blue to green represents the level of fatty acids in decreasing order. (**B**) Identification of principal component analysis (PCA) of Raw, Germ, Ferm (*L. reuterii* AKT1) and G+F was shown by comparing PC1 with PC2 as well as PC1 with PC3. Germ- germinated brown rice, Ferm- fermented brown rice, G+F- germinated + fermented brown rice.

In the present study, seventeen fatty acids were detected in differently treated brown rice samples (Table S5), and as shown in Table S5, fatty acid levels were found to be significantly different in all four differently treated samples (*p* ≤ 0.05). The results show that the highest levels of fatty acids were found in fermented brown rice. In particular, fermentation has been indicated as a tool to enhance the nutritional value of foods, both in terms of the increased bioavailability of bioactive compounds and in terms of the production of health-promoting end-products. Among the latter, short-chain fatty acids (SCFAs) have emerged as some of the most studied compounds in the last decade, due to their proven benefit. Heat map analysis was used for separating fatty acids based on different concentration, represented from blue to green in decreasing order (Figure 5A). Fatty acids like stearic acid [55], linoleic acid [56], valeric acid [57], lauric acid [58] and azelaic acid [59] were already reported in the literature as strong antioxidants and stress-reducing compounds. Principal component analysis (PCA) was used to understand the correlation among different groups, and we found that the fermented brown rice sample (Ferm *L. reuterii* AKT1) was divergent from the other three samples. Moreover, germinated and G+F BR were found to be correlated (Figure 5B) (Table S5).

Additionally, to understand the correlations and diversity among differently treated samples, as well as for the better elucidation of results, PCA was also performed by combining all the analyses together (amino, phenolic, organic and fatty acids) (Figure S2). In joint PCA, fermented BR was found to be divergent from the other three samples, similar to that which we have observed in all separate analyses. Additionally, Germ BR and Raw BR were found to be correlated with each other.

Hence, by comparing different approaches in our study, it is clear that fermentation increases most of the bioactive compounds in cereal grains like brown rice [60].

#### 3.4.5. Peptides Identified in Brown Rice Samples

An alternative, less expensive method for producing bioactive peptides from protein substrates is microbial fermentation. The main advantage of using microbes instead of enzymes is that appropriately used microorganisms will not only break down proteins into peptides and free amino acids, but can also remove anti-nutritional or hyper-allergenic factors that may be present (such as phytate, stachyose, saponins, etc.) [61,62]. In the present study, most of the peptides were reported in fermented brown rice samples (*L. reuterii* AKT1) (Table 2). Therefore, we analyzed the peptides produced in the different fermented and germinated samples and a total of 10 known peptides were found, and their potential functions were determined by comparing them with similar peptides already reported in the literature (Table 2). Most of the peptides identified in our study were not known in the databases so it is difficult to predict their functions. These bioactive peptides reported in our study in the fermented samples contribute to the strong antioxidative and stress-reducing abilities. In the meantime, functional analysis is required to confirm the antioxidative and stress-related abilities of peptides in the fermented sample containing sequences of already reported peptides.

## 4. Conclusions

In our research, we identified that fermented brown rice exhibited high antioxidant activities as well as a significant amount of phenolic compounds and flavonoids, as compared to raw, germination and germination combined with fermentation samples. This might be because, when germination and fermentation were combined, biological enzymes utilized bioactive compounds as their energy sources. Additionally, using untargeted metabolomics, we found that *L. reuterii* fermented brown rice enhanced the production of essential amino acids and organic acids. The fermented samples also enhanced phenolic compounds and fatty acids, and were found to be rich in antioxidant and stress-reducing peptides. These findings have shown that fermented *L. reuterii* AKT1 brown rice consumption can provide optimal health benefits, as it is rich in antioxidant phytonutrients. Moreover, these findings also make this sample a promising material for the development of functional food with great antioxidant and stress-reducing abilities. However, further studies concerning the ability of *L. reuterii* AKT1 fermented brown rice to reduce stress-related disorders require in-vivo studies for further confirmation of their health-promoting effects and functional food development.

## Figures and Tables

**Table 1:** Total antioxidants, DPPH, ABTS and FRAP, total phenolic content (TPC) and total flavonoid content (TFC) of different rice samples.

S.NO	Sample	DPPH (mg Trolox Equivalent 100 g, DW)	ABTS (mg Trolox Equivalent 100 g, DW)	FRAP (mg Trolox Equivalent 100 g, DW)	TPC (mg Gallic Acid Equivalent 100 g, DW)	TFC (mg Catechin Equivalent 100 g, DW)
1	Raw BR	25.653 ± 0.98 ^a^	21.86 ± 0.93 ^a^	19.21 ± 2.64 ^a^	16.75 ± 0.75 ^a^	14.42 ± 0.80 ^a^
2	Germinated BR	89.602 ± 1 ^b^	98.37 ± 1.38 ^b^	88.04 ± 1.27 ^b^	74.70 ± 0.72 ^b^	62.88 ± 2.62 ^b^
3	Fermented BR	114.40 ± 0.66 ^d^	130.52 ± 0.97 ^d^	111.16 ± 1.83 ^d^	97.13 ± 0.59 ^d^	79.62 ± 1.33 ^d^
4	G+F BR	105.99 ± 0.59 ^c^	110.11 ± 0.26 ^c^	96.61 ± 2.29 ^c^	89.94 ± 0.25 ^c^	69.85 ± 0.80 ^c^

BR—brown rice, G+F—germination combined with fermentation. Results are expressed as mean ± SD of triplicate analyses. Different alphabetical letters (^a–d^) in each column represent statistically significant differences (Tukey and Duncan test, *p* ≤ 0.05). DW—dry weight sample.

**Table 2:** Identification of peptides in different processed brown rice samples.

S.No	Sample Name	Retention Time	Peak Area	Adduct/Charge	Precursor Mass	Found at Mass	Formula Finder Result	Peptide	Role	Reference
1	Raw	0.9	3.92 × 10^2^	[M+H]+	229.155	229.1556	C_11_H_20_N_2_O_3_	Leucylproline (Leu-Pro)	Reduction in Stress/Depression And Antioxidant	[63]
	Germ	1.4	2.34 × 10^5^	[M+H]+	229.155	229.1553				
	Ferm	1.41	5.18 × 10^3^	[M+H]+	229.155	229.1552				
	G+F	1.4	1.02 × 10^5^	[M+H]+	229.155	229.1552				
2	Raw	18.52	2.98 × 10^4^	[M+Na]+	525.287	525.2886	C_14_H_26_N_4_O_6_	Gln-Ser-Leu Glutaminyl-seryl-leucine	Antioxidant	[64]
	Germ	18.51	1.52 × 10^5^	[M+Na]+	525.287	525.2892				
	Ferm	18.51	2.31 × 10^5^	[M+Na]+	525.287	525.2891				
	G+F	18.51	1.60 × 10^5^	[M+Na]+	525.287	525.2890				
3	Raw	24.01	1.15 × 10^4^	[M+NH4]+	445.288	445.2884	C_18_H_33_N_7_O_5_	Gly-Pro-Arg-Val	Sleep inducing peptide	[65]
	Germ	24.01	9.03 × 10^3^	[M+Na]+	450.244	450.2448				
	Ferm	24.01	9.88 × 10^6^	[M+Na]+	450.244	450.2446				
	G+F	23.98	1.18 × 10^5^	[M+Na]+	450.244	450.2441				
4	Raw	ND	ND	ND	ND	ND	C_12_H_25_N_5_O_3_	Arg-leu	Antioxidant	[66]
	Germ	ND	ND	ND	ND	ND				
	Ferm	1.29	1.89 × 10^3^	[M+H]+	288.203	288.2027				
	G+F	ND	ND	ND	ND	ND				
5	Raw	ND	ND	[M−H]−	ND	ND	C_11_H_20_N_2_O_5_	Glu-Leu	Antioxidant	[67]
	Germ	ND	ND	[M−H]−	ND	ND				
	Ferm	10.08	3.79 × 10^3^	[M−H]−	259.131	259.1294				
	G+F	0	0	[M−H]−	0	0				
6	Raw	ND	ND	[M+CH3OH+H]+	ND	ND	C_7_H_12_N_2_O_3_	gly-pro	Stress reduction	[68]
	Germ	1.26	9.30 × 10^4^	[M+CH3OH+H]+	205.118	205.1187				
	Ferm	1.18	1.51 × 10^3^	[M+CH3OH+H]+	205.118	205.1193				
	G+F	1.39	6.27 × 10^3^	[M+CH3OH+H]+	205.118	205.1193				
7	Raw	ND	ND	[M+H]+	ND	ND	C_10_H_17_N_3_O_6_S	L.gamma.glu-L.cystein (Glutathione)Peptide	Antioxidant	[69]
	Germ	ND	ND	[M+H]+	ND	ND				
	Ferm	1.82	1.08 × 10^4^	[M+H]+	437.163	437.1693				
	G+F	ND	ND	[M+H]+	ND	ND				
8	Raw	ND	ND	[M+H]+	ND	ND	C_14_H_20_N_4_O_7_S	Gly-Gly-Gly	Antioxidant	[70]
	Germ	ND	ND	[M+H]+	ND	ND				
	Ferm	40.08	1.08 × 10^4^	[M+H]+	389.113	389.1127				
	G+F	ND	ND	[M+H]+	ND	ND				
9	Raw	16.03	1.23 × 10^4^	[M−H]−	186.115	186.1144	C_9_H_17_NO_3_	Pivagabine (N-pivaloyl-γ-aminobutyric acid)	Stress and Anxiety	[71]
	Germ	16.02	8.71 × 10^3^	[M−H]−	186.115	186.1139				
	Ferm	16.04	6.83 × 10^5^	[M−H]−	186.115	186.1138				
	G+F	16.04	3.15 × 10^5^	[M−H]−	186.115	186.1138				
10	Raw	47.27	6.72 × 10^5^	[M−H]−	459.196	459.1957	C_16_H_28_N_8_O_8_	Gly-Arg-Gly-Asp-Gly	Peripheral Nerve Regeneration and Antioxidant	[72]
	Germ	47.26	7.26 × 10^5^	[M−H]−	459.196	459.196				
	Ferm	47.28	8.73 × 10^5^	[M−H]−	459.196	459.1959				
	G+F	47.27	9.41 × 10^5^	[M−H]−	459.196	459.1958				

Germ- germinated brown rice, Ferm- fermented brown rice, G+F- germinated + fermented brown rice.

## Data Availability

Not required.

## Supplementary Materials

The following are available online at https://www.mdpi.com/article/10.3390/antiox10040626/s1. Figure S1: Germinated brown rice. Figure S2: Principal component analysis (PCA) of Raw, Germ, Ferm (*L. reuterii* AKT1) and G+F were shown by comparing component 1(PC1) with component 2 (PC2). Table S1: GABA concentration detected by HPLC in Raw, different lactic acid bacteria fermented brown rice, germinated, and germination combined with fermentation (G+F). Table S2: Amino acids detected in different processed brown rice samples (raw, germinated, fermented (*L. reuterii* AKT1), and germinated+ fermented (*L. reuterii* AKT1). Table S3: Phenolic componds detected in different processed brown rice samples (raw, germinated, fermented (*L. reuterii* AKT1), and germinated+ fermented (*L. reuterii* AKT1). Table S4: Organic acids detected in different processed brown rice samples (raw, germinated, fermented (*L. reuterii* AKT1), and germinated+ fermented (*L. reuterii* AKT1). Table S5: Fatty acids detected in different processed brown rice samples (raw, germinated, fermented (*L. reuterii* AKT1), and germinated+ fermented (*L. reuterii* AKT1).

## Abbreviations

BR	Raw brown rice
Germ	Germinated brown rice
Ferm/Ferm (*L. reuterii*)	Fermented brown rice
G+F/G+F (*L. reuterii*)	Germinated combined with fermented brown rice
