# Supplementary material for: Untargeted Metabolomics of Korean Fermented Brown Rice Using UHPLC Q-TOF MS/MS Reveal an Abundance of Potential Dietary Antioxidative and Stress-Reducing Compounds
Source: Antioxidants (Basel). 2021 Apr 19;10(4):626. doi: 10.3390/antiox10040626 (PMC8072674; doi:10.3390/antiox10040626)
Supplement: Supplementary file 1 [file antioxidants-10-00626-s001.zip › antioxidants-1132165-supplementary.pdf]

Table S1: GABA concentration detected by HPLC in Raw, different lactic acid bacteria fermented brown rice, germinated, and germination combined with fermentation (G+F)

S.No	Sample	Area	Concentration	Retention time (Min.)
1	Raw brown rice	20.65584	1.611±0.001[a]	12.695
2	*P.pentosaceus* (FMC1) Fermented brown rice	53.30829	6.24±0.057[d]	12.712
3	*L.fermentum* (FMF2) Fermented brown rice	59.30758	7.03±0.055[e]	12.71
4	*L.fermentum* (AKT2) Fermented brown rice	192.989	19.07±0.069[h]	12.843
5	*L.rhamnosus* (FMR2) Fermented brown rice	83.40036	8.03±0.058[f]	12.815
6	*L.rhamnosus* (FMR1) Fermented brown rice	46.32564	5.27±0.045[b]	12.855
7	*L.brevis* (FMB1) Fermented brown rice	52.21344	6.19±0.064[d]	12.856
8	*L.brevis* ATCC (STANDARD) Fermented brown rice	53.56727	6.28±0.069[d]	12.856
9	*L.plantarum* (FMP1) Fermented brown rice	50.30663	5.80±0.010[c]	12.862
10	*L.plantarum* (FMP2) Fermented brown rice	51.5759	10.04±0.069[g]	12.866
11	*L.reuterii* (AKT1) Fermented brown rice	275.2176	27.03±0.055[j]	12.865
12	Germinated brown rice	304.6879	34.40±0.026[k]	12.508
13	G+F (*L.reuterii*) brown rice	220.83539	22.91±0.028[i]	12.509

All values are expressed as the mean ± SD of triplicate experiments. The samples concentration used was 1mg/ml and a-k superscripts with different letters indicate a significant difference while sample superscript letters indicate no significant difference (Tukey and Duncan test p ≤ 0.05) DW, dry weight sample.

Table S2: Amino acids detected in different processed brown rice samples (raw, germinated, fermented (*L.reuterii* AKT1), and germinated+ fermented (*L.reuterii* AKT1).

S.No	Sample Name	Retention time (Min)	Peak Area	Adduct/ Charge	Precursor mass	Found at mass	Formula finder result	Amino acid
1	Raw	1	1.92E+03±0.2[a]	[M+H]+	156.077	156.077	$C_6H_9N_3O_2$	Histidine
	Germ	1.01	1.11E+05±0.36[b]	[M+H]+	156.077	156.0772		
	Ferm	1	6.60E+05±0.76[c]	[M+H]+	156.077	156.077		
	G+F	1	7.21E+05±0.55[d]	[M+H]+	156.077	156.077		
2	Raw	ND	ND	[M-H]-	ND	ND	$C_6H_{14}N_2O_2$	Lysine
	Germ	0.97	4.59E+03±0.52[a]	[M-H]-	145.099	145.0986		
	Ferm	1.02	1.64E+06±0.28[c]	[M-H]-	145.099	145.0984		
	G+F	1	9.86E+05±0.50[b]	[M-H]-	145.099	145.0983		
3	Raw	ND	ND	[M+H]+	ND	ND	$C_5H_{11}NO_3S$	Methionine
	Germ	1.17	7.04E+04±0.55[a]	[M+H]+	166.053	166.0535		
	Ferm	1.18	2.49E+05±0.52[c]	[M+H]+	166.053	166.0538		
	G+F	1.17	2.20E+05±0.50[b]	[M+H]+	166.053	166.0538		
4	Raw	1.17	2.89E+02±0.76[a]	[M-H]-	146.047	146.0457	$C_5H_9NO_4$	Glutamic Acid
	Germ	1.49	2.15E+03±0.51[b]	[M-H]-	146.047	146.0457		
	Ferm	1.49	1.32E+06±0.55[d]	[M-H]-	146.047	146.046		
	G+F	1.5	9.27E+05±0.55[c]	[M-H]-	146.047	146.0459		
5	Raw	ND	ND	[M+H]+	ND	ND	$C_4H_9NO_2$	Gamma-Aminobutyric Acid
	Germ	1.1	7.46E+05±1.00 [c]	[M+H]+	104.071	104.0705		
	Ferm	1.16	7.10E+05±0.55 [b]	[M-H]-	102.057	102.0565		
	G+F	1.18	2.76E+04±0.52 [a]	[M-H]-	102.057	102.0563		
6	Raw	ND	ND	[M+H]+	ND	ND	$C_6H_{14}N_4O_2$	Arginine
	Germ	1.02	4.64E+06±0.55[b]	[M+H]+	175.118	175.1184		
	Ferm	1.11	5.99E+06±0.5[c]	[M+H]+	175.118	175.1182		
	G+F	1.33	5.92E+04±0.50[a]	[M+H]+	175.118	175.1182		
7	Raw	ND	ND	[M-H]-	ND	ND	$C_5H_{11}NO_2$	Valine
	Germ	ND	ND	[M-H]-	ND	ND		
	Ferm	1.47	9.27E+05±0.51[b]	[M-H]-	116.073	116.0719		
	G+F	1.48	4.60E+05±0.50[a]	[M-H]-	116.073	116.0716		
8	Raw	1.14	1.19E+03±0.36[a]	[M-H]-	132.031	132.0307	$C_4H_7NO_4$	Aspartic acid
	Germ	ND	ND	[M-H]-	ND	ND		
	Ferm	1.13	5.26E+05±0.46[c]	[M-H]-	132.031	132.0302		
	G+F	1.13	2.99E+05±0.50[b]	[M-H]-	132.031	132.0303		

9	Raw	ND	ND	[M+H]+	ND	ND	$C_9H_{11}NO_2$	Phenylalanine
	Germ	4	1.86E+05±1.0[a]	[M+H]+	166.086	166.086		
	Ferm	4.03	1.97E+06±0.51[b]	[M+H]+	166.086	166.0865		
	G+F	4	3.44E+06±0.52[c]	[M+H]+	166.086	166.0858		
10	Raw	ND	ND	[M-H]-	ND	ND	$C_5H_{12}N_2O_2$	Ornithine
	Germ	0.99	3.19E+03±0.51[a]	[M-H]-	131.084	131.0829		
	Ferm	1.02	6.72E+05±0.55[c]	[M-H]-	131.084	131.0828		
	G+F	1	4.72E+05±0.50[b]	[M-H]-	131.084	131.0827		
11	Raw	1.12	3.31E+02±0.15[a]	[M-H]-	104.036	104.0353	$C_3H_7NO_3$	Serine
	Germ	1.11	3.52E+03±0.51[b]	[M-H]-	104.036	104.0356		
	Ferm	1.11	6.33E+04±0.55[d]	[M-H]-	104.036	104.0354		
	G+F	1.11	3.94E+04±0.50[c]	[M-H]-	104.036	104.0355		
12	Raw	ND	ND	[M-H]-	ND	ND	$C_6H_{13}NO_2$	Leucine
	Germ	2.46	3.57E+03±0.60[a]	[M-H]-	130.088	130.0875		
	Ferm	2.43	6.31E+06±0.55[c]	[M-H]-	130.088	130.0874		
	G+F	2.45	2.67E+06±0.51[b]	[M-H]-	130.088	130.0874		
13	Raw	ND	ND	[M-H]-	ND	ND	$C_5H_{10}N_2O_3$	Glutamine
	Germ	1.12	5.92E+03±0.64[a]	[M-H]-	145.063	145.0619		
	Ferm	1.12	6.32E+03±0.55[b]	[M-H]-	145.063	145.063		
	G+F	1.12	1.47E+05±0.52[c]	[M-H]-	145.063	145.062		
14	Raw	ND	ND	[M-H]-	ND	ND	$C_9H_{11}NO_3$	Tyrosine
	Germ	2.07	9.70E+03±0.60[a]	[M-H]-	180.068	180.0672		
	Ferm	1.91	3.19E+04±0.55[c]	[M-H]-	180.068	180.0669		
	G+F	1.95	3.02E+04±0.50[b]	[M-H]-	180.068	180.0668		
15	Raw	ND	ND	[M-H]-	ND	ND	$C_4H_9NO_3$	Threonine
	Germ	ND	ND	[M-H]-	ND	ND		
	Ferm	1.11	1.10E+04±0.55[a]	[M-H]-	118.052	118.0513		
	G+F	1.95	3.02E+04±0.50[b]	[M-H]-	118.052	180.0668		
16	Raw	ND	ND	[M-H]-	ND	ND	$C_4H_8N_2O_3$	Asparagine
	Germ	ND	ND	[M-H]-	ND	ND		
	Ferm	1.11	2.97E+05±0.55[b]	[M-H]-	131.047	131.0461		
	G+F	1.11	1.94E+05±0.51[a]	[M-H]-	131.047	131.0462		
17	Raw	ND	ND	[M-H]-	ND	ND	$C_{11}H_{12}N_2O_2$	Tryptophan
	Germ	ND	ND	[M-H]-	ND	ND		
	Ferm	7.62	2.84E+06±0.55[b]	[M-H]-	203.084	203.0829		
	G+F	7.65	1.20E+06±0.52[a]	[M-H]-	203.084	203.0828		

18	Raw	ND	ND	[M+H]+	ND	ND	$C_5H_9NO_2$	Proline
	Germ	1.15	1.01E+06±0.80[b]	[M+H]+	130.05	130.0499		
	Ferm	1.17	8.81E+06±0.55[c]	[M+H]+	130.05	130.0499		
	G+F	1.15	5.60E+05±0.55[a]	[M+H]+	130.05	130.0501		

Results are expressed as mean ± SD of triplicate analyses. Different alphabetical letters in each column represent statistically significant differences (Tukey and Duncan test p ≤ 0.05) DW, dry weight sample

Table S3: Phenolic componds detected in different processed brown rice samples (raw, germinated, fermented (*L.reuterii* AKT1), and germinated+ fermented (*L.reuterii* AKT1).

S.No	Sample Name	Retention time	Peak Area	Adduct/Charge	Precursor mass	Found at mass	Formula finder result	Phenolic compound
1	Raw	45.5	8.87E+04±0.52[a]	[M+H]+	353.268	353.2847	$C_{25}H_{36}O$	Beta-carotenol
	Germ	Nd	Nd	[M+H]+	Nd	Nd		
	Ferm	45.49	9.18E+04±0.5[b]	[M+H]+	353.268	353.2848		
	G+F	Nd	Nd	[M+H]+	Nd	Nd		
2	Raw	Nd	Nd	[M+H]+	Nd	Nd	$C_{10}H_{12}O_2$	Eugenol
	Germ	20.85	2.58E+04±0.51[a]	[M+H]+	179.107	179.1067		
	Ferm	20.85	2.12E+05±0.50[c]	[M+H]+	179.107	179.1069		
	G+F	20.83	1.11E+05±0.50[b]	[M+H]+	179.107	179.1068		
3	Raw	33.8	2.10E+06±1.00[a]	[M-H]-	293.177	293.1761	$C_{17}H_{26}O_4$	6-Gingerol
	Germ	33.79	2.10E+06±0.25[c]	[M-H]-	293.177	293.176		
	Ferm	33.82	2.15E+06±0.51[d]	[M-H]-	293.177	293.1762		
	G+F	33.81	2.07E+06±1.0[b]	[M-H]-	293.177	293.176		
4	Raw	Nd	Nd	[M-H]-	Nd	Nd	$C_{15}H_{10}O_4$	Chrysin
	Germ	Nd	Nd	[M-H]-	Nd	Nd		
	Ferm	14.78	4.09E+05±0.50[a]	[M-H]-	253.052	253.0509		
	G+F	Nd	Nd	[M-H]-	Nd	Nd		
5	Raw	Nd	Nd	[M-H]-	Nd	Nd	$C_{16}H_8N_2O_5$	Apigenin
	Germ	Nd	Nd	[M-H]-	Nd	Nd		
	Ferm	14.78	4.34E+05±0.5[a]	[M-H]-	269.047	269.0456		
	G+F	Nd	Nd	[M-H]-	Nd	Nd		
6	Raw	Nd	Nd	[M+H]+	Nd	Nd	$C_9H_6O_2$	Coumarin
	Germ	1.89	2.20E+05±0.50[b]	[M+H]+	147.044	147.0444		
	Ferm	1.87	1.24E+05±0.51[a]	[M+H]+	147.044	147.0447		
	G+F	1.92	2.94E+05±0.51[c]	[M+H]+	147.044	147.0444		
7	Raw	12.31	ND	[M+H]+	305.071	305.067	$C_{15}H_{14}O_7$	Epigallocatechin
	Germ	12.3	4.51E+04±0.51[a]	[M+H]+	305.071	305.067		
	Ferm	12.29	1.44E+06±1.0[c]	[M+H]+	305.071	305.067		
	G+F	12.26	5.98E+05±0.60[b]	[M+H]+	305.071	305.067		

8	Raw	Nd	Nd	[M+H]+	Nd	Nd	$C_7H_{19}N_3$	Spermidine
	Germ	0.94	5.55E+04±0.51[a]	[M+H]+	188.176	188.1761		
	Ferm	0.96	1.33E+06±0.52[c]	[M+H]+	188.176	188.1761		
	G+F	0.95	1.96E+05±0.55[b]	[M+H]+	188.176	188.1761		
9	Raw	38.06	4.13E+05±0.50[a]	[M-H]-	277.182	277.1812	$C_{17}H_{26}O_3$	6-Paradol
	Germ	38.04	4.25E+05±0.50[b]	[M-H]-	277.182	277.1813		
	Ferm	38.08	4.38E+05±0.51[d]	[M-H]-	277.182	277.1811		
	G+F	38.07	4.36E+05±0.52[c]	[M-H]-	277.182	277.1811		
10	Raw	Nd	Nd	[M-H]-	Nd	Nd	$C_9H_8O_2$	Cinnamic acid
	Germ	Nd	Nd	[M-H]-	Nd	Nd		
	Ferm	4	1.10E+06±1.02[b]	[M-H]-	147.046	147.0452		
	G+F	4.01	4.10E+05±0.55[a]	[M-H]-	147.046	147.0453		
11	Raw	Nd	Nd	[M+H]+	Nd	Nd	$C_9H_8O_3$	p-Coumaric acid
	Germ	Nd	Nd	[M+H]+	Nd	Nd		
	Ferm	1.89	1.35E+06±0.50[a]	[M+H]+	182.081	182.0811		
	G+F	Nd	Nd	[M+H]+	Nd	Nd		
12	Raw	Nd	Nd	[M-H]-	Nd	Nd	$C_9H_{10}O_3$	Methoxyphenylacetic acid
	Germ	Nd	Nd	[M-H]-	Nd	Nd		
	Ferm	15.28	1.27E+07±1.0[b]	[M-H]-	165.057	165.0556		
	G+F	15.28	4.04E+06±1.0[a]	[M-H]-	165.057	165.0557		
13	Raw	Nd	Nd	[M-H]-	Nd	Nd	$C_7H_6O_3$	Sesamol/ 2-Hydroxybenzoic acid
	Germ	Nd	Nd	[M-H]-	Nd	Nd		
	Ferm	19.63	1.35E+05±0.51[a]	[M-H]-	137.025	137.025		
	G+F	Nd	Nd	[M-H]-	Nd	Nd		
14	Raw	Nd	Nd	[M-H]-	Nd	Nd	C_8H_8O	Vanillic acid/ Acetophenone
	Germ	Nd	Nd	[M-H]-	Nd	Nd		
	Ferm	15.28	5.64E+06±1.0[b]	[M-H]-	119.051	119.0504		
	G+F	15.29	5.01E+06±0.5[a]	[M-H]-	119.051	119.0503		

Results are expressed as mean ± SD of triplicate analyses. Different alphabetical letters in each column represent statistically significant differences (Tukey and Duncan test $p \leq 0.05$) DW, dry weight sample

Table S4: Organic acids detected in different processed brown rice samples (raw, germinated, fermented (*L.reuterii* AKT1), and germinated+ fermented (*L.reuterii* AKT1).

S.No	Sample Name	Retention time	Peak Area	Adduct/Charge	Precursor mass	Found at mass	Formula finder result	Organic acid
1	Raw	31.12	2.80E+05±0.50[a]	[M+H]+	445.12	445.1193	$C_4H_6O_5$	Malic acid
	Germ	32.04	1.21E+06±1.0[b]	[M+H]+	445.12	445.1194		
	Ferm	52.87	7.40E+06±1.0[d]	[M+H]+	445.12	445.1193		
	G+F	52.83	3.45E+06±0.60[c]	[M+H]+	445.12	445.1195		
2	Raw	39.74	4.44E+05±1.0[a]	[M+H]+	205.086	205.0864	$C_{12}H_{12}O_3$	Anofinic acid
	Germ	ND	ND	[M+H]+	ND	ND		
	Ferm	39.76	7.02E+05±0.52[c]	[M+H]+	205.086	205.0865		
	G+F	39.74	5.84E+05±1.0[b]	[M+H]+	205.086	205.0864		
3	Raw	ND	ND	[M+H]+	ND	ND	$C_8H_{14}O_2S_2$	Lipoic acid
	Germ	1	1.05E+05±0.55[b]	[M+H]+	207.051	207.0512		
	Ferm	1.01	2.15E+04±0.52[a]	[M+H]+	207.051	207.0504		
	G+F	ND	ND	[M+H]+	ND	ND		
4	Raw	ND	ND	[M+H]+	ND	ND	$C_5H_6O_4$	Itaconic acid
	Germ	1.18	1.27E+05±1.01[a]	[M+H]+	148.061	148.0606		
	Ferm	1.21	1.83E+06±1.0[c]	[M+H]+	148.061	148.0608		
	G+F	1.16	7.14E+05±0.50[b]	[M+H]+	148.061	148.0608		
5	Raw	ND	ND	[M+H]+	ND	ND	$C_9H_8O_3$	4-Hydroxycinnamic acid/ P-coumaric acid
	Germ	ND	ND	[M+H]+	ND	ND		
	Ferm	1.89	1.35E+06±1.0[a]	[M+H]+	182.081	182.0811		
	G+F	ND	ND	[M+H]+	ND	ND		
6	Raw	1.2	1.28E+04±1.01[a]	[M-H]-	195.052	195.0513	$C_6H_{12}O_7$	Gluconic acid
	Germ	1.2	2.49E+05±1.0[b]	[M-H]-	195.052	195.0511		
	Ferm	1.19	6.07E+05±0.51[d]	[M-H]-	195.052	195.0513		
	G+F	1.2	3.37E+05±0.64[c]	[M-H]-	195.052	195.051		
7	Raw	ND	ND	[M-H]-	ND	ND	$C_3H_4O_4$	Malonic acid
	Germ	1.21	1.05E+03±0.50[a]	[M-H]-	103.005	103.0036		
	Ferm	1.23	1.33E+06±1.01[c]	[M-H]-	103.005	103.0038		
	G+F	1.23	9.74E+05±0.60[b]	[M-H]-	103.005	103.0038		
8	Raw	ND	ND	[M-H]-	ND	ND	$C_5H_{10}O_6$	Arabinonic Acid
	Germ	ND	ND	[M-H]-	ND	ND		

	Ferm	1.21	7.39E+05±0.54[b]	[M-H]-	165.041	165.0409		
	G+F	1.21	6.24E+05±1.0[a]	[M-H]-	165.041	165.0406		
9	Raw	ND	ND	[M-H]-	ND	ND	$C_4H_6O_4$	Succinic acid
	Germ	2.23	3.98E+03±0.64[a]	[M-H]-	117.02	117.0199		
	Ferm	1.39	3.38E+05±1.0[c]	[M-H]-	117.02	117.0195		
	G+F	2.22	1.38E+05±0.68[b]	[M-H]-	117.02	117.0194		
10	Raw	ND	ND	[M-H]-	ND	ND	$C_9H_{17}NO_8$	Neuraminic acid
	Germ	ND	ND	[M-H]-	ND	ND		
	Ferm	1.13	2.15E+05±0.68[b]	[M-H]-	266.089	266.0882		
	G+F	1.15	7.14E+03±1.0[a]	[M-H]-	266.089	266.0884		
11	Raw	ND	ND	[M-H]-	ND	ND	$C_8H_6O_4$	Isophthalic Acid
	Germ	ND	ND	[M-H]-	ND	ND		
	Ferm	1.76	7.24E+05±0.72[b]	[M-H]-	199.039	199.038		
	G+F	1.78	3.90E+05±0.80[a]	[M-H]-	199.039	199.036		
12	Raw	ND	ND	[M+H]+	ND	ND	$C_5H_{11}N_3O_2$	Butanoic acid (Guanidinobutanoic acid)
	Germ	1.34	8.15E+03±1.0[a]	[M+H]+	146.092	146.0922		
	Ferm	1.35	3.69E+05±0.64[c]	[M+H]+	146.092	146.0923		
	G+F	1.34	2.01E+05±0.68[b]	[M+H]+	146.092	146.0923		
13	Raw	ND	ND	[M+H]+	ND	ND	$C_{12}H_{21}NO_6$	glutaric acid (O-Glutarylcarnitine)
	Germ	2.51	5.71E+04±0.50[a]	[M+H]+	276.145	276.1445		
	Ferm	2.53	3.51E+06±0.60[c]	[M+H]+	276.145	276.1442		
	G+F	2.55	8.74E+05±0.81[b]	[M+H]+	276.145	276.1448		
14	Raw	2.2	1.78E+03±1.0[a]	[M-H]-	154.975	154.9745	$C_5H_4N_2S_2$	Pyrazinoic acid
	Germ	31.53	5.18E+03±0.52[b]	[M-H]-	154.975	154.9743		
	Ferm	47.13	3.89E+04±0.76[c]	[M-H]-	154.975	154.9745		
	G+F	47.13	4.62E+04±0.72[d]	[M-H]-	154.975	154.9745		
15	Raw	ND	ND	[M+H]+	ND	ND	$C_6H_8O_6$	Ascorbic acid (Vitamin C)
	Germ	ND	ND	[M+H]+	ND	ND		
	Ferm	1.02	4.95E+03±0.80[a]	[M+H]+	209.009	209.0107		
	G+F	ND	ND	[M+H]+	ND	ND		
16	Raw	ND	ND	[M+H]+	ND	ND	$C_6H_5NO_2$	Nicotinic acid (vitamin B3)
	Germ	1.75	2.58E+05±0.95[b]	[M+H]+	124.039	124.0395		
	Ferm	1.78	4.95E+05±0.64[c]	[M+H]+	124.039	124.0392		
	G+F	1.75	2.47E+04±0.80[a]	[M+H]+	124.039	124.0396		

17	Raw	ND	ND	[M-H]-	ND	ND	$C_4H_8O_3$	Hydroxybutyric acid
	Germ	ND	ND	[M-H]-	ND	ND		
	Ferm	3.04	7.10E+05±0.72[a]	[M-H]-	103.041	103.04		
	G+F	ND	ND	[M-H]-	ND	ND		
18	Raw	ND	ND	[M-H]-	ND	ND	$C_7H_{10}O_7$	Homocitric acid
	Germ	ND	ND	[M-H]-	ND	ND		
	Ferm	1.03	2.18E+05±0.85[a]	[M-H]-	205.036	205.036		
	G+F	ND	ND	[M-H]-	ND	ND		
19	Raw	ND	ND	[M-H]-	ND	ND	$C_{18}H_{32}O_5$	Malyngic acid
	Germ	ND	ND	[M-H]-	ND	ND		
	Ferm	30.87	4.38E+05±1.0[a]	[M-H]-	327.219	327.2181		
	G+F	ND	ND	[M-H]-	ND	ND		

Results are expressed as mean ± SD of triplicate analyses. Different alphabetical letters in each column represent statistically significant differences (Tukey and Duncan test p ≤ 0.05) DW, dry weight sample

Table S5: Fatty acids detected in different processed brown rice samples (raw, germinated, fermented (*L.reuterii* AKT1), and germinated+ fermented (*L.reuterii* AKT1).

S.No	Sample Name	Retention time	Peak Area	Adduct/ Charge	Precursor mass	Found at mass	Formula finder result	Fatty acid
1	Raw	23.74	2.64E+04±1.02[a]	[M-H]-	239.061	239.0597	$C_8H_{16}O_2$	Octanoic acid
	Germ	30.93	3.72E+04±0.52[b]	[M-H]-	239.061	239.0594		
	Ferm	31.29	4.92E+04±0.50[d]	[M-H]-	239.061	239.0595		
	G+F	31.22	4.46E+04±1.01[c]	[M-H]-	239.061	239.0594		
2	Raw	28.79	6.01E+03±0.55[a]	[M-H]-	255.234	255.2331	$C_{16}H_{32}O_2$	Palmitic Acid
	Germ	39.95	2.00E+05±1.01[c]	[M-H]-	255.234	255.2332		
	Ferm	39.98	2.33E+05±0.50[d]	[M-H]-	255.234	255.233		
	G+F	32.29	4.14E+03±0.50[b]	[M-H]-	255.234	255.2331		
3	Raw	ND	ND	[M+H]+	ND	ND	$C_5H_{10}O_2$	Valeric acid
	Germ	ND	ND	[M+H]+	ND	ND		
	Ferm	22.88	1.88E+04±0.50[a]	[M+H]+	185.066	185.0663		
	G+F	ND	ND	[M+H]+	ND	ND		
4	Raw	46.24	5.29E+05±1.05[a]	[M-H]-	279.234	279.2332	$C_{18}H_{32}O_2$	Linoleic Acid
	Germ	46.23	5.48E+05±1.0[b]	[M-H]-	279.234	279.2331		
	Ferm	46.25	5.86E+05±1.0[c]	[M-H]-	279.234	279.2336		
	G+F	46.25	6.68E+05±0.52[d]	[M-H]-	279.234	279.2333		
5	Raw	47.28	1.35E+06±1.0[a]	[M+H]+	271.264	271.2637	$C_{17}H_{34}O_2$	Heptadecanoic acid
	Germ	47.27	1.48E+06±0.50[c]	[M+H]+	271.264	271.2638		
	Ferm	47.29	1.56E+06±1.01[d]	[M+H]+	271.264	271.2637		
	G+F	47.27	1.45E+06±1.0[b]	[M+H]+	271.264	271.2635		
6	Raw	27.69	5.28E+03±1.04[a]	[M-H]-	283.265	283.2644	$C_{18}H_{36}O_2$	Stearic acid
	Germ	28.19	6.67E+03±1.0[b]	[M-H]-	283.265	283.2644		
	Ferm	28.57	8.46E+03±0.40[d]	[M-H]-	283.265	283.2643		
	G+F	27.79	6.78E+03±1.0[c]	[M-H]-	283.265	283.2644		
7	Raw	39.15	5.15E+05±1.07[a]	[M+H]+	200.201	200.2015	$C_{12}H_{24}O_2$	Lauric acid
	Germ	39.16	5.17E+05±0.50[b]	[M+H]+	200.201	200.2015		
	Ferm	39.16	5.50E+05±0.50[d]	[M+H]+	200.201	200.2015		
	G+F	39.14	5.33E+05±0.51[c]	[M+H]+	200.201	200.2014		
8	Raw	34.57	1.21E+04±0.52[a]	[M-H]-	243.161	243.1605	$C_{13}H_{24}O_4$	

	Germ	34.56	3.19E+05±0.51[c]	[M-H]-	243.161	243.1605		Tridecanedioic acid
	Ferm	34.59	3.21E+05±0.50[d]	[M-H]-	243.161	243.1606		
	G+F	34.58	2.96E+05±0.52[b]	[M-H]-	243.161	243.1605		
9	Raw	ND	ND	[M+H]+	ND	ND	$C_{12}H_{20}O_3$	Traumatin
	Germ	32.83	6.34E+03±0.52[a]	[M+H]+	213.149	213.1488		
	Ferm	32.82	1.71E+06±0.6[c]	[M+H]+	213.149	213.1491		
	G+F	32.8	4.42E+05±0.55[b]	[M+H]+	213.149	213.1491		
10	Raw	ND	ND	[M-H]-	ND	ND	$C_{18}H_{32}O_5$	Octadecadienoic acid/ Corchorifatty acid F
	Germ	ND	ND	[M-H]-	ND	ND		
	Ferm	30.87	4.38E+05±0.51[a]	[M-H]-	327.219	327.2181		
	G+F	ND	ND	[M-H]-	ND	ND		
11	Raw	ND	ND	[M-H]-	ND	ND	$C_{18}H_{34}O_3$	Ricinoleic acid
	Germ	ND	ND	[M-H]-	ND	ND		
	Ferm	2.32	1.35E+03±0.51[a]	[M-H]-	297.12	297.2425		
	G+F	ND	ND	[M-H]-	ND	ND		
12	Raw	ND	ND	[M-H]-	ND	ND	$C_6H_{12}O_4$	Mevalonic Acid
	Germ	ND	ND	[M-H]-	ND	ND		
	Ferm	3.48	2.72E+05±0.52[b]	[M-H]-	147.067	147.0666		
	G+F	3.49	1.19E+05±0.52[a]	[M-H]-	147.067	147.0666		
13	Raw	22.59	9.99E+04±0.50[a]	[M-H]-	187.099	187.0979	$C_9H_{16}O_4$	Azelaic Acid
	Germ	22.47	7.23E+04±0.50[b]	[M-H]-	187.099	187.098		
	Ferm	22.51	6.28E+05±0.52[d]	[M-H]-	187.099	187.0978		
	G+F	22.51	4.78E+05±0.50[c]	[M-H]-	187.099	187.0978		
14	Raw	ND	ND	[M-H]-	ND	ND	$C_9H_{18}O_3$	9-Hydroxynonanoic acid
	Germ	ND	ND	[M-H]-	ND	ND		
	Ferm	23.49	7.46E+04±0.51[b]	[M-H]-	173.119	173.1186		
	G+F	23.48	5.29E+04±1.0[a]	[M-H]-	173.119	173.1186		
15	Raw	ND	ND	[M-H]-	ND	ND	$C_{19}H_{36}O_5$	Bempedoic acid
	Germ	ND	ND	[M-H]-	ND	ND		
	Ferm	26	1.84E+05±0.50[a]	[M-H]-	407.193	407.193		
	G+F	ND	ND	[M-H]-	ND	ND		
16	Raw	39.06	1.45E+05±0.50[a]	[M-H]-	313.24	313.2389	$C_{18}H_{34}O_4$	

	Germ	39.05	2.69E+05±1.1[b]	[M-H]-	313.24	313.2387		Octadecanedioic acid
	Ferm	39.07	3.52E+05±0.50[c]	[M-H]-	313.24	313.2388		
	G+F	39.06	4.04E+05±0.50[d]	[M-H]-	313.24	313.2389		
17	Raw	ND	ND	[M-H]-	ND	ND	$C_{18}H_{34}O_5$	Pinellic acid
	Germ	34.31	2.75E+04±0.52[a]	[M-H]-	329.234	329.2339		
	Ferm	32.84	8.55E+06±0.5[c]	[M-H]-	329.234	329.233		
	G+F	32.83	7.83E+06±1.0[b]	[M-H]-	329.234	329.2331		

Results are expressed as mean ± SD of triplicate analyses. Different alphabetical letters in each column represent statistically significant differences (Tukey and Duncan test p ≤ 0.05) DW, dry weight sample

Figure S1: Germinated brown rice:

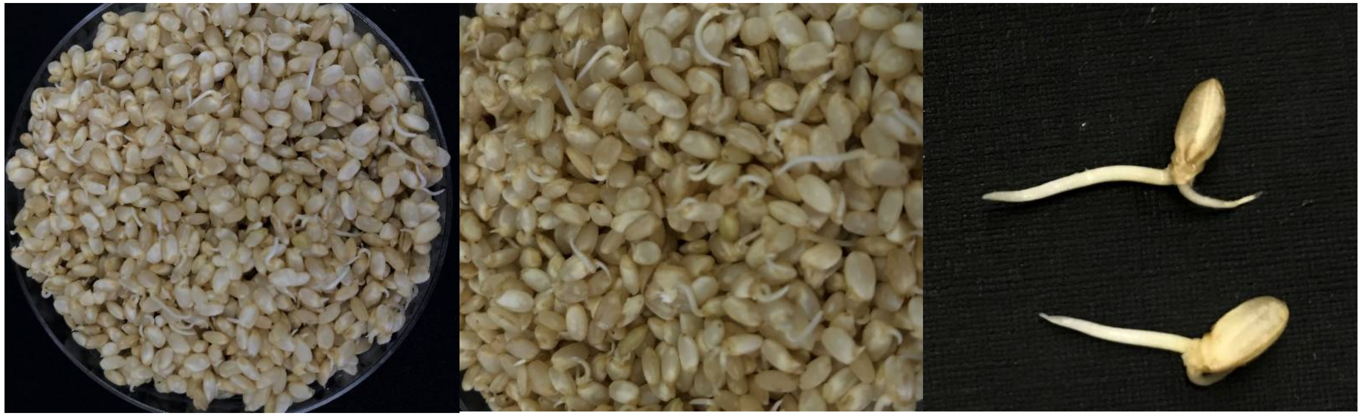

Figure S2: Principal component analysis (PCA) of Raw, Germ, Ferm (*L. reuterii* AKT1) and G+F were shown by comparing component 1(PC1) with component 2 (PC2)

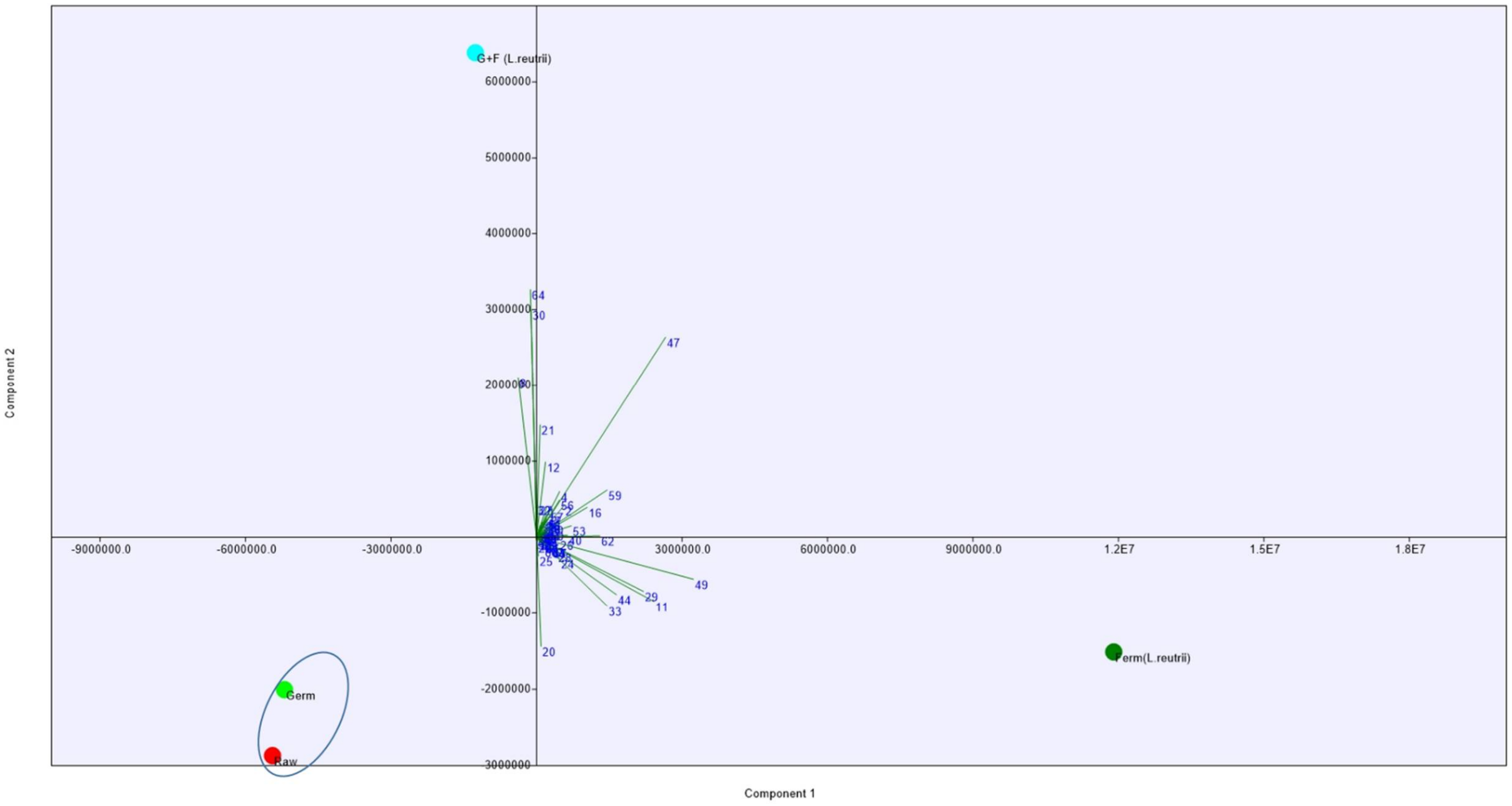

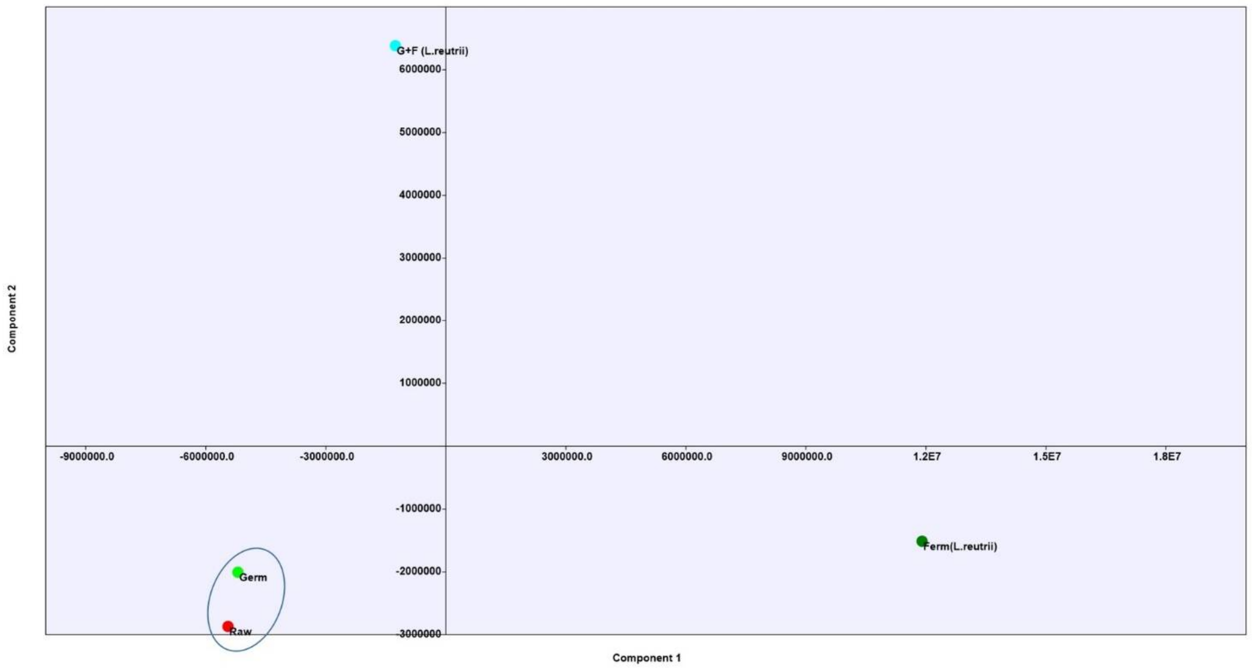

Figure S2 A). Identification of principal component analysis (PCA) of raw, germ, ferm (*L. reuterii*) and G+F were shown by comparing PC 1 with PC2 with Biplot. Where 1 to 68 number represents different bioactive compounds (amino acids, phenolic compounds, organic acids and fatty acids). B). Identification of principal component analysis (PCA) of raw, germ, ferm (*L. reuterii*) and G+F were shown by comparing PC 1 with PC2 without Biplot.

Amino acid (1-18) -(1-Histidine, 2-Lysine, 3-Methionine, 4-Glutamic acid, 5-arginine, 6-Valine, 7-Aspartic acid, 8-Phenylalanine, 9-Ornithine, 10-Serine, 11-Leucine, 12-Glutamine, 13-Tyrosine, 14-Threonine, 15-Asparagine, 16-Tryptophan, 17-Proline, 18-Gamma-aminobutyric acid)

Phenolic compounds (19-32)- (19- Eugenol, 20- 6-Gingerol, 21- Chrysin, 22- Coumarin, 23- Apigenin, 24- spermidine, 25- 6-Paradol, 26- Epigallocatechin, 27-p-Coumaric acid, 28-Cinnamic acid, 29-Methoxyphenylacetic, 30- 2-Hydroxybenzoic acid, 31- Vanillic acid, 32- β-carotenol)

Fatty acid (33-49)- (33- Octanoic acid, 34- Palmitic Acid, 35- Linoleic acid, 36 Heptadecanoic acid, 37- Stearic acid, 38- Lauric acid, 39- Tridecanedioic acid, 40- Traumatin, 41- Octadecadienoic acid/ Corchorifatty acid F, 42- Ricinoleic acid, 43- Mevalonic Acid, 44- Valeric acid, 45- Bempedoic acid, 46- Azelaic Acid, 476- Hydroxynonanoic Acid, 48- Octadecanedioic acid, 49- Pinellic acid)

Organic acid (50-68) (50- Malic acid, 51- Anofinic acid, 52- Lipoic Acid, 53- Itaconic acid, 54- Gluconic acid, 55- Succinic acid, 56- Malonic acid, 57- Arabinonic Acid, 58- Butanoic acid, 59- Neuraminic acid, 60- Isophthalic Acid, 61- Malyngic acid, 62- Glutaric acid, 63- Hydroxybutyric acid, 64- pyrazinoic acid, 65- Ascorbic acid, 66- Nicotinic acid, 67- p-Coumaric acid, 68- Homocitric acid)
